# Towards reimagined technical assistance: the current policy options and opportunities for change

**DOI:** 10.12688/gatesopenres.13204.1

**Published:** 2020-12-16

**Authors:** Alexandra Nastase, Alok Rajan, Ben French, Debarshi Bhattacharya

**Affiliations:** 1 Oxford Policy Management, Oxford, UK; 2 Bill and Melinda Gates Foundation, Delhi, India

**Keywords:** international development, technical assistance, capacity development, capacity substitution, capacity supplementation, policy options, state capability

## Abstract

Technical assistance has been at the heart of development assistance provided to country governments by donor agencies over the past several decades. The current debates on reimagining technical assistance focus on the existing challenges of the different types of technical assistance, and the (re)construction of an ideal model for delivering this type of support, with little discussion about the dilemmas involved in making day-to-day decisions and trade-offs in implementation. This article presents technical assistance as a policy option for governments and details the existing models of delivering technical assistance, as well as their limitations and the required enabling conditions. The models presented focus on the type of role for the technical advisers- as doers (performing government functions), partners (working with the government to perform a specific role) and facilitators (enabling and facilitating change programmes to address wicked problems). As part of the programmes, the team of technical advisers can play one or more roles. The authors also discuss how problem-solving or solution implementation can be the focus of a technical assistance programme or how large programmes combine these two approaches. The paper provides a practical account of the implications of the programme design that would ideally help governments and donors to design more effective technical assistance programmes.

## Disclaimer

The views expressed in this article are those of the author(s). Publication in Gates Open Research does not imply endorsement by the Gates Foundation.

## Introduction

Technical assistance has been at the heart of development assistance provided to country governments by donor agencies over the past several decades. Many cross-organisational initiatives of the international development community recognise the need to reimagine the models of technical assistance to support country development goals more effectively. There are a variety of approaches that define a new wave of technical assistance, including thinking and working politically ( TWP CoP, 2013), development entrepreneurship ( Faustino & Booth, 2014), problem-driven ietartive adaptation ( Andrews *et al.*, 2012), adaptive management practices ( USAID, 2016), the Child Health Task Force in Nigeria ( Child Heath Task Force, 2019) or the Coaching Approach ( Cashin, 2020). Most of these models share key principles, such as including local actors, focusing on problems rather than solutions, working as part of systems, and allowing space for course correction during implementation, to mention just a few.

The current focus of these debates seems to be on the existing challenges of the different types of technical assistance and the (re)construction of an ideal model for delivering this type of support. There is less discussion about the dilemmas involved in making day-to-day decisions and trade-offs in implementation. The degree of success achieved in implementing these key principles to improve development outcomes is not yet documented in a strong evidence base ( Laws & Marquette, 2018), and we believe that the reasons behind this need to be made part of any discussion of the challenges and trade-offs that governments, donors, and implementing partners face when trying to apply these principles. 

This paper argues that the discussion on technical assistance needs to be re-framed as a government policy choice. It describes the options available at the moment and discusses the practical implications of this framework for technical assistance programming.

## Re-framing technical assistance as a government policy option

The premise of the paper is that technical assistance is a domestic policy option. Governments make decisions every day to balance short-term political agendas with long-term development objectives. To achieve these objectives, governments need support. Strengthening government capacity is not an agenda only for the less developed countries; it is an ongoing agenda for all governments. The main difference is that some countries may need support from development partners to finance and sometimes co-create some of the technical assistance they require.

The same type of support is available, no matter who finances it. Development partners can play one or more roles at the same time, including as doers (substituting government capacity), partners (supporting governments, usually in areas of highly specialised expertise), or facilitators (supporting complex change programmes to strengthen state capability). In this paper, we define capacity substitution as technical assistance that is focused on performing functions that the government is expected to perform. While we use capacity supplementation to refer to technical assistance that is focused on complementing government’s efforts or providing unique technical expertise. Capacity development refers to technical assistance that is provided in a facilitation role, and it focuses on strengthening the government’s capacity to deliver the functions it is supposed to deliver.

In practice, technical assistance programmes employ a solution-driven (start with the solution and find ways to implement it) or a problem-driven approach (start with the problem and find ways to address the problem). From our experience, technical programmes can be designed by strictly choosing one approach, other large programmes especially treat these options as a continuum and move across the continuum depending on the context and the issues to be addressed.

To choose from the right type or to combine different types of technical assistance, the government needs to be clear about the objectives of the support they require, and hopefully also to link this with their vision for the country’s development priorities. For instance, if governments choose to use externals to do the work and replace government functions, it is not realistic to expect that this will build the capability to do the work independently of consultants. On the contrary, it will create a need for more consultancy services in the future to deliver that function. Similarly, partnerships to deliver work may be ideal to fill essential gaps at critical moments for a government, but the success of building capacity will be rather contextual and rarely intentional as the support is focused on the outputs and less on the process of learning.

There is an in-principle agreement in the development community that problem-driven support is more effective than solution-driven technical assistance ( Sparrow, 2008), ( Andrews *et al.,* 2012). However, the practice is diverse. The main advantage of a problem-driven approach is that it focuses on salient public sector problems, as opposed to assistance that follows the agenda of an external donor. The solution-driven approach risks to lack ownership by the local government.

**Figure 1.**
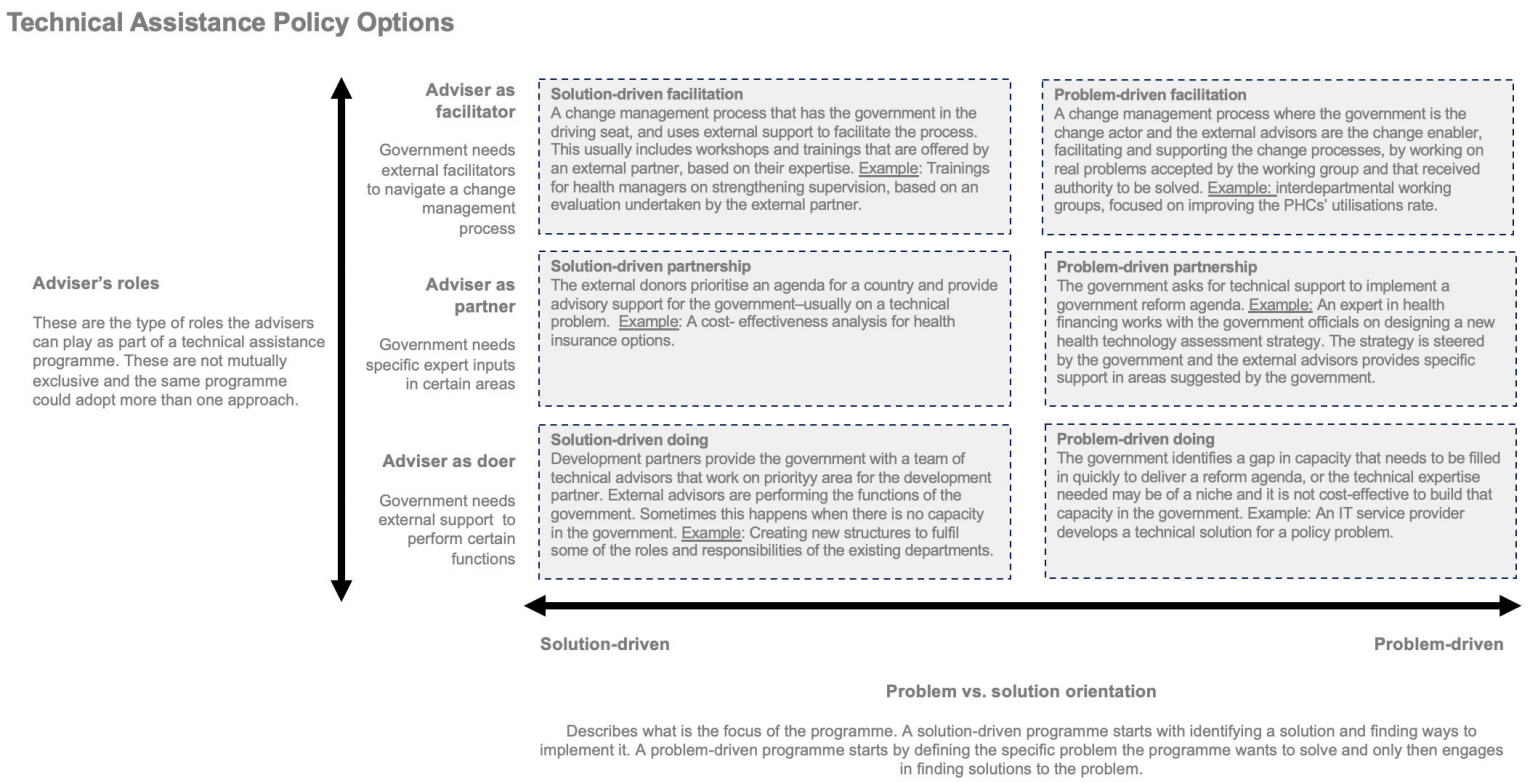
Technical assistance policy options. Each box represents a technical assistance model. Six boxes are presented on two axis – the adviser’s role and the problem orientation of the programme.

The framework presented in Figure 1 sets out this policy decision as being based on two options: the type of technical assistance activity and problem orientation. This is not a normative framework guiding how technical assistance should be delivered; it is an empirical framework for how capacity development is currently delivered, focusing on the options governments currently face.

Government, funders, and the providers of technical assistance must ensure that due attention is invested in building this shared (and honest) view of the problems that the support is seeking to tackle. Special attention is warranted in suitably breaking down visions or long-term impact goals into tangible and realistic outputs and milestones. Ring-fencing these issues during the design stage, and subsequently developing and agreeing on the appropriate rules of engagement between the parties, is key to ensuring that the support remains focused on the core issues and can over time build sustainable capacity in the recipient government. This process requires a meaningful and equal dialogue between governments and funders when designing technical assistance programmes.

## Problem orientation

At the conceptual level, a solution-driven technical assistance programme would start by identifying an approach and advocate for its implementation in different contexts. By contrast, a technical assistance programme that is problem-driven would begin with the government defining the problem and only then move to testing and implementing solutions. In practice, this involves more of a dialogue between governments and development partners. Fundamentally, the vision of the government and its aspirations define the problems and the choice of technical assistance. The rest of this section explores the options available to governments.

Historically, many technical assistance and capacity development programmes have been solution-driven, with development partners focused on solutions that can be applied across geographies. Driven by global definitions and frameworks, it is easy for solution-driven technical assistance to overwhelm the national institutions quickly, and sometimes to either mute local voices or affect the capacity of governments to articulate their problems.

**Figure 2.**
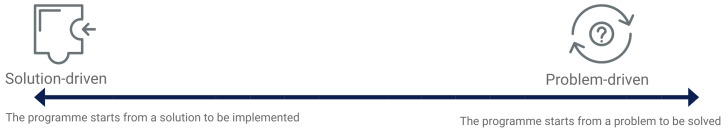
Problem orientation. Each icon represents a type of problem orientation, one is solution-driven and the other is problem-driven. The arrow shows that these may be placed on a continuum as part of the same programme and are, in practice, not mutually exclusive.

There is increasing consciousness in the larger international development community of the need to adopt a problem-driven approach to delivering technical assistance. Governments, and the issues they face, exist in their own unique context, and this results in varying capacity gaps. It is recognised that recipients themselves are best placed to identify and articulate these capacity gaps. Figure 2 below shows that, in practice, the government may choose from a continuum of problem and solution-driven approaches as part of the same technical assistance programme, depending on their needs.

## Roles for the technical advisers

**Figure 3.**
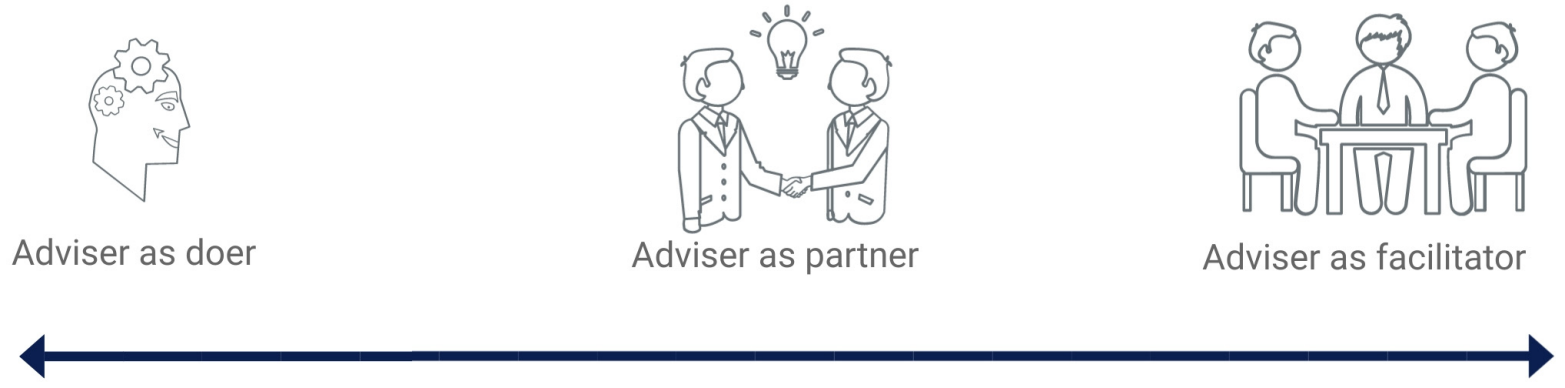
Adviser’s roles. Each icon represents a type of role for the technical advisers: doer, partner, facilitator. The arrow shows that these may be placed on a continuum as part of the same programme and are, in practice, not mutually exclusive.

As the government may have varied problems to address, an effective technical assistance programme can take various shapes. Technical assistance programmes need to be set up to address the core issues of the host government. The goal for governments and funders alike should be to decide the main objective of the technical assistance and to choose amongst a variety of roles for technical advisers, taking account of the limitations and the enabling conditions for each approach. The same programme may require a combination of different types of inputs, from short trainings delivered by specialist consultants to change processes facilitated by externals that may require intensive engagement from the government and the externals, at multiple levels and over many years. In practice, the objectives may not be easy to isolate and therefore Figure 3 below shows the type of roles for the technical advisors as not mutually exclusive – for instance, the same team may include both facilitators and partners.

## Adviser as doer

A common model is for the technical advisers to be doers, by which, for a variety of reasons, they perform government functions. This model is usually linked to capacity substitution or in-sourcing. At least two types of scenarios are seen in practice.

First, the government needs to perform specific functions but cannot perform them. This may refer to not having staffing or technical competencies. Sometimes, there may be an urgent request and the process of acquiring the inputs (mostly technical) needs to be expedited. At other times, the capacity needed may refer to a niche that the government would not require beyond the current assignment, so they decide it is not good value for money to build the capacity in-house. For example, government agencies often rely on IT firms to develop internal systems and platforms – which are largely one-off events – that are then delivered to and used by the host department.

Capacity substitution is usually deliberately applied in cases where:

•   the primary purpose of the support is not capacity development, but the delivery of certain predefined results;

•   there is an understanding of how the deliverables will fit into the broader system and an open channel to work with other government departments impacted by the work of the technical adviser; and/or

•   there is a lack of in-house technical expertise, which is needed urgently or that may not make sense to develop in-house in the long run.

This model is frequently used in practice when the government or the donor is impatient to get results while cutting through red tape. As such, while there may be some value in this model for specific resourcing gap-filling needs, by design, this model has clear limitations in building state capability. Furthermore, the model can have severe consequences for state capability, when the objectives are not clear and the technical advisors end up performing the core functions of the government, such as regulation, provision, funding, or service delivery. Table 1 below explores in detail the characteristics of this type of technical assistance, including its limitations.

**Table 1.** Three approaches to technical assistance.

	Capacity substitution	Capacity supplementation	Capacity development
**Role for the** **advisor**	DOER, performing government functions	PARTNER, providing specific support to the government (in most cases, technical support on specialised areas)	FACILITATOR, working with government to enable change and facilitate complex processes
**Policy need**	The government needs specific inputs but does not have the capacity to provide them – sometimes the need is urgent and no capacity is available, at other times the capacity needed is a niche that the government would not invest in beyond the current assignment so they in-source. • Clear technical problem • Known solution/output • Capacity constraints that cannot be filled with existing resources	The government needs specific expert inputs in certain areas. • Moderately sophisticated problems • Specific technical inputs required • Government requires additional support but leads the process	The government needs support in implementing a complex change process, and it requires specialised support to facilitate the process. • Complex change management problems • It is not clear what type of support is required • Support in identifying problems to solve, and testing different solutions, is required • The government will be involved and in the driving seat
**Programme** **characteristics**	• Clearly defined outputs to be delivered and reported against • Led by external actors with control from the government counterparts • Limited or no learning, the programme is mainly focusing on outputs • The advisors are expected to have technical competencies, and to be local or international experts.	• Predefined outputs but also outcome-based technical support • Led by the government counterparts or external actors • Learning depends on the context, the implementing partner, and it is not necessarily embedded in the programme design. • The advisors are expected to have both technical and professional skills, sometimes including facilitation skills in a specific area. In most cases, the expectation for the experts is to have international expertise.	• Outcomes-based programmes, with flexible and adaptive programme framework - regularly updated to navigate existing spaces for reform. • Led by the government and facilitated by external actors. • The role of the advisor is to manage a process, rather than the content of the programme. • Learning is central to programme design – both real-time learning for course correction as well as learning about what works, what doesn’t and why in that particular context. • The advisors can be both local and international, and the type of competencies required refer primarily to their facilitation skills and capacity to work with senior leaders and bureaucrats – although technical skills and experience in the area of work will be appreciated.
**Enabling** **conditions**	• Clearly identified gap in existing capacity • High levels of acceptance that there is a capacity gap to be filled • Some open channels to other government departments impacted by the work of the technical advisor • Authorisation to work on filling the capacity gap	• Medium levels of authority to engage on the subject • Medium levels of acceptance, ability, and willingness to partner with the advisor on the subject • An initial identification of areas of support needed	• High levels of acceptance that there is a problem to be solved/ something needs to be done • Authority, ideally from the highest political levels, to have the government teams engaged in the change process • At least medium level of ability of the government team to work on the change process
**Key risks**	• Lack of acceptance at various levels in the government (sometimes, a principal may request support but others in government may feel threatened or just not accept that the support is needed) • Lack of authority in government (the person who opts for this activity may lose authority or may not have authority over the relevant departments) • When this is based on supply, rather than demand, it may end up duplicating efforts and damaging the current reform efforts	• Lack of engagement from the government: sometimes the government may ask the advisor to lead the process, which eventually damages the level of ownership of the results • No authority in government • When this is based on supply rather than demand, it may provide highly technical advice, but not grounded in the local political economy • No follow-through after the end of the programme/ the funding period • No capacity developed, given a limited involvement from the government counterparts • Unintended consequences of diverting resources from other reform areas	• Having the support of a recognised authority in government is critical to undertake this type of activity. Relying too much on only one • person may damage the success of the programme • Losing the interest of the principal, for instance by focusing on too many long- term results and not enough quick wins to create acceptance • Focusing too much on developing technical skills and not understanding the political economy • Serious gaps in terms of hardware, infrastructure, supply chains, staff available • Unintended consequences of diverting resources from other reform areas

The methods of delivering this type of technical assistance vary. Sometimes programmes may involve niche technical support, such as building IT infrastructure; fellowships/consultancies of international or nationals working in host countries to deliver independently on behalf of the government; or reports, strategies etc. prepared by the external experts with no involvement of the government staff.

## Adviser as partner

This model is usually linked to capacity supplementation. The government needs are also primarily technical and relate to the previously identified gap areas. The premise is that the government is already performing certain functions, but requires specific inputs in certain challenging areas, or needs to bring in newer and better ways of working from the outside. This model of technical assistance is deliberately applied in cases where:

•   the primary purpose of the support is to deliver outputs, but also transfer knowledge to the host government;

•   the need for support is limited and clearly identified and the technical assistance providers are able to bring in that specific expertise; and/or

•   the government is looking to bring in support in specific areas that are challenging but that are already being delivered by the government possibly with sub-optimal quality and/or efficiency, or introducing different and innovative ways of producing the desired output.

Many small to medium-sized development projects fall under this category. Governments make the best use of these type of support when it is demand-driven, and when the inputs required from the externals specifically contribute to solving a problem they are tackling. For example, in certain Indian states, the departments of environment and climate change bring in local academic institutions and technical firms to execute highly technical work, such as co-developing international funding proposals for climate adaptation projects. Similarly, multiple state-level departments of industry often engage professional consulting firms to support their private investment promotion wings and to bring more professional and corporate ways of working, which can work better with potential private investors.

The methods of delivering this type of technical assistance vary. Most frequently, this includes delivering outputs such as studies, reports, strategies, analysis by working with government counterparts. It can also include fellowships – when the fellows are working with governments on a specific project for a set timeframe. Often, external technical assistance aimed at capacity supplementation might need to demonstrate the effectiveness of the new ways of working and/or innovations being introduced to the government by ‘doing’ them themselves. However, the temporary or tactical nature of this activity distinguishes this type of technical assistance from the previous type (substitution/in-sourcing).

## Adviser as facilitator

There seems to be a consensus that for technical assistance that is focused on behaviour and systems change, a strongly facilitator-based approach is necessary ( Le *et al.,* 2016). Models of technical assistance that explicitly build long-term capacity in the government rely heavily on having the government counterparts in the driving seat and the advisor as merely a facilitator and change manager. While such a model can be used for reform processes of varying complexity, it is most valuable when embarking on sophisticated and complex change processes. The role of the facilitator is usually to help the government navigate the various stages of the change management process and to challenge the way of doing things.

•   This model of technical assistance is usually applied in cases where:

•   the primary purpose of the support is to develop long-lasting capacity at individual, organisational, and institutional levels in the host government;

the government and the development partner are ready to invest time in building capacity sustainably – it will take longer to see the results, but there is a higher chance that these will be sustainable;

•   the need for support relates to change-facilitation, or behaviour and systems change, and the specific outcomes or change pathways are not clearly defined or identified;

•   the nature of the problem is highly dynamic, fluid, and ever-changing, and requires significant changes in the hardware and software elements of organisations; and/or

•   the process for reaching the end result is not always clearly defined, and the government is not able to exercise full control over the reform process as there are many systemic challenges that influence the reform process.

Multiple methods have been applied to develop this type of approach in the past few years, including: problem-driven iterative adaptation (facilitating a process through which the government team engages in solving problems), mentorship (using recognised external experts in particular fields to provide guidance on specific development objectives), and coaching (using external coaches to help individuals achieve the objectives, by providing a motivating environment and by challenging current ways of working, thinking etc). Fellowships can also be found in this category if they work with nationals and focus on supporting their development as part of the systems they are working in. Since the typical view of technical assistance supporting the government is long term, structural reforms in core public delivery processes and sub-systems are attempted in this type of assistance (e.g. public financial management systems, data/management information systems, performance management systems, and human resources policies).

## The implications of these policy options for the programming led by development partners

Our framework presents the current policy options for governments when choosing to use technical assistance to achieve their objectives. From our experience, there are many aspects governments must balance in their decision-making process. This section aims to highlight a few of those we have frequently witnessed in practice.

Technical assistance is a policy option. Every day, governments make decisions that balance the short-term political agenda with long-term development objectives. To achieve these objectives, governments need support. Strengthening government capacity is not an agenda only for the least developed countries, it is an ongoing agenda for all governments. The main difference is that poorer countries may need support from international donors to design and finance some of the technical assistance they require.

The same types of support are available, no matter who finances it: doer, partner, facilitator. Governments need to know what they need and how that will fit in their vision for the country. The most common fallacy is to expect every type of technical assistance to lead to capacity development. We do not believe that is the case. Suppose governments choose to use externals to do the work and replace government functions. In that case, it is not realistic to expect that it will build a capability to do the work independently of consultants.

On the contrary, it will create a case for more consultancy services in the future. Similarly, partnerships to deliver work may have practical limitations as regards building capacity, but they can fill essential gaps at critical moments for a government. Capacity development means enabling national actors to deliver functions they are designed to deliver. This takes time, patience, resources, consistency, and complementarity. It goes beyond the life of a programme, and can require donors to come together in a joint effort to support the country’s development objectives.

The main challenge to framing technical assistance as a policy option may come from the perceived balance of power between governments and donors. The more invested the donors are, the more power they can have in influencing the reform agenda. In cases where they provide substantial financial support to national reform programmes, it may be difficult for some government counterparts to negotiate their need for technical assistance. The most daunting consequence may be for government counterparts to feel disempowered as regards making a choice about technical assistance, and instead of seeing this as a building block to accessing the required financial support.

Demand-driven or solution-driven technical assistance. Based on years of experience, there is an agreement in the development community that demand-driven support is more effective than solution-driven technical assistance. Its main advantage is that it focuses on salient problems, as opposed to an external donor agenda, which may lack ownership by the local government, and suffer from a lack of drive by the government. However, development partners often invest in programmes for which there is not a clear demand from the government, and they are sometimes ‘tolerated’ or even welcomed in doing so.

This apparent dichotomy in the perceived problem statements is the key consideration in developing a technical assistance engagement that is genuinely effective and responsive. Government, funders, and the providers of technical assistance must ensure that due attention is invested in building this shared (and honest) view of the problems that the support is seeking to tackle. Ring-fencing these issues during the design stage, and subsequently developing and agreeing on the appropriate rules of engagement between the parties, is key to ensuring that the support remains focused on the core issues, and can over time build sustainable capacity in the recipient government. This process requires a meaningful and equal dialogue between governments and funders in the design of technical assistance programmes.

In practice, an important consideration is balancing the government needs in terms of capacity. As mentioned in the previous section, governments – especially in low-resource environments – may have different needs as regards getting things done. They also need to balance the short-term political cycles with the long-term institutional reforms’ agenda. Sometimes they need specialised support to solve a technical problem (which we termed capacity substitution), at other times they may need high-level challenging functions to support them to achieve their objectives (which we termed capacity development). With clarity about the merits and limits of each approach, a large multi-year technical assistance programme for system strengthening may successfully draw on different types of support at various times. There are two potential tensions: on the one hand, using one type of support (for instance, capacity substitution) – hoping it will lead to something that it is not designed to do (for instance, capacity development). The other tension is that governments and donors alike feel they need to show quick results at home: governments need to show results to their citizens and donors need to show the results of using taxpayer money. This may result in less patience as regards focusing on capacity development and processes, and more impatience as regards addressing issues without a long-term view of building institutional capacity.

Building the right teams to deliver capacity development. Historically, there has been a prioritisation of technical skills above other skills, such as interpersonal skills, understanding of political context, and relatable expertise. As the role of technical assistance providers gradually shifts from pure implementation to more facilitation, it will be important to engage individuals who play that convening role, in addition to technical leadership. It is important to engage advisors who can build trust with counterparts, and that can communicate and network effectively. At the same time, power may need to shift from international to national experts. This is easier said than done. A few challenges may need to be overcome. For instance, the current power structures favour international technical experts, including as part of the donor organisations and implementing partners. Additionally, there is an accountability trap of focusing on high-quality results and less on building processes to support capacity development in the government and the larger ecosystem – including local consultants and external local organisations, who are likely to be present there after the end of the support.

Departmental ownership as a priority. Departmental ownership is essential for effective reform but is very tough to achieve in practice. It is important to recognise that, at times, a ‘yes’ may not mean a yes in practice, and implementers should manage their expectations accordingly. Recipient agencies are often so dependent on donors for the bulk of their funding and/or functional resources that they hesitate to oppose any suggestion by a donor, even when government officials feel it is not in the country’s best interest. In such a scenario, government officials tend to engage superficially in the technical assistance effort, without real ownership. The technical assistance providers need to have the guile to be able to tell the difference, but also to prioritise the approaches that have the government’s buy-in. At the same time, pulling our support when there is no interest or uptake may be a valid policy option as well, although less exercised in practice.

Learn from new ways of delivering technical assistance. The challenges posed by the lack of evidence on the effectiveness of technical assistance have already been discussed. Donors and their implementation partners can do more to create incentives structures for self-reflection and learning, ideally documenting both successes and failures and their enabling factors. With more development agendas are driven by national interests, multilateral and donor organisations may need to step up and enhance the risk appetite for learning from experimentation to solve wicked problems.

## Data and availability

No data are associated with this article.

## References

[ref-1] Andrews M Pritchett L Woolcock M : Escaping Capability Traps through Problem Driven Iterative Adaptation (PDIA) - CID Working Paper No. 240.Boston: Center for International Development, Harvard University. 2012. Reference Source

[ref-2] Cashin C : The Coaching Approach: Changing the way development assistance is done.Online:2020; Accessed on September 2020. Reference Source

[ref-9] Child Health Task Force: Reimagining Technical Assistance. Nigeria Status Update. 2019. Reference Source

[ref-3] Faustino J Booth J : Development entrepreneurship – how donors and leaders can foster institutional change. 2014. Reference Source

[ref-4] Laws E Marquette H : Thinking and working politically. Reviewing the evidence on the integration of politics into development practice over the past decade. 2018. Reference Source

[ref-5] Le LT Anthony BJ Bronheim SM : A Technical Assistance Model for Guiding Service and Systems Change. *J Behav Health Serv Res.* 2016;43(3):380–95. 10.1007/s11414-014-9439-2 25239308

[ref-6] Sparrow M : The Character of Harms: Operational Challenges in Control.New York: Cambridge University Press. 2008. Reference Source

[ref-7] TWP CoP: So what does ‘thinking and working politically’ look like? 2013. Reference Source

[ref-8] USAID: ADS Chapter 201 - Program Cycle Operational Policy. 2016. Reference Source

