# Towards reimagined technical assistance: the current policy options and opportunities for change

**DOI:** 10.12688/gatesopenres.13204.2

**Published:** 2021-10-18

**Authors:** Alexandra Nastase, Alok Rajan, Ben French, Debarshi Bhattacharya

**Affiliations:** 1 Oxford Policy Management, Oxford, UK; 2 Bill and Melinda Gates Foundation, Delhi, India

**Keywords:** international development, technical assistance, capacity development, capacity substitution, capacity supplementation, policy options, state capability

## Abstract

Technical assistance has been at the heart of development assistance provided to country governments by donor agencies over the past several decades. The current debates on reimagining technical assistance focus on the existing challenges of the different types of technical assistance and the (re)construction of an ideal model for delivering this type of support, with little discussion about the dilemmas involved in making day-to-day decisions and trade-offs in implementation. This article presents technical assistance as a policy option for governments and details the existing models of delivering technical assistance, their limitations, and the required enabling conditions. The models presented focus on the type of role for the technical advisers- as doers (performing government functions), partners (working with the government to perform a specific role) and facilitators (enabling and facilitating change programmes to address wicked problems). Finally, the paper provides a practical account of the implications of the programme design and suggests potential opportunities for change particularly in the context of COVID-19 pandemic. It complements an open letter on the practical account of the current challenges in the design and implementation of technical assistance programmes.

## Disclaimer

The views expressed in this article are those of the author(s). Publication in Gates Open Research does not imply endorsement by the Gates Foundation.

## Introduction

Technical assistance has been at the heart of development assistance provided to country governments by donor agencies over the past several decades. Many cross-organisational initiatives of the international development community recognise the need to reimagine the models of technical assistance to support country development goals more effectively. There are a variety of approaches that define a new wave of technical assistance, including thinking and working politically ( TWP CoP, 2013), development entrepreneurship ( Faustino & Booth, 2014), problem-driven iterative adaptation ( Andrews *et al*., 2012), adaptive management practices ( USAID, 2016), the Child Health Task Force in Nigeria ( Child Health Task Force, 2019) or the Coaching Approach ( Cashin, 2020) to name some of the most prominent. Most of these models share key principles, such as including local actors, focusing on problems rather than solutions, working as part of systems, and allowing space for course correction during implementation. The degree of success achieved in implementing these fundamental principles to improve development outcomes is not yet documented in a solid evidence base ( Laws & Marquette, 2018). We provide a brief review of these approaches and their principles of implementation in our complimentary article on reimagining technical assistance ( Nastase *et al*., 2020)

This paper discusses technical assistance as a government policy option to strengthen policymaking or build state capability. It provides a positive framework of analysis that includes current options, their advantages, and their limitations. Additionally, the paper also explores the implications of each approach on policy and programme management and reflections on opportunities for change, including opportunities arising from COVID-19 pandemic responses.

## Re-framing technical assistance as a government policy option

We refer to technical assistance as non-financial support, usually knowledge-based, contracted by and/or provided to governments by local or international experts to support policymaking and/or strengthen state capability. There are significant differences in the types of technical assistance provided, based on several dimensions. In this section, we aim to capture some of the main differences.

First, technical assistance can differ through the way it is funded: from sovereign funds coming from the recipient of technical assistance, to public funds directed through development support, from bilateral agreements (e.g. the UK Foreign and Commonwealth Development Office or the Australian Department for Foreign Assistance and Trade etc.), multilateral organisations (the United Nations, the World Bank or the International Monetary Fund) or from supranational regional bodies (the European Commission). Alternatively, funding can also come from philanthropies (e.g. Bill and Melinda Gates Foundation etc.) or Non-Governmental Organisations (Red Cross etc.) The technical assistance can come in different shapes, for instance as part of a lending programme usually tied to performance indicators, or under the form of reimbursable or non-reimbursable funds, as well as grants to external advisors directly.

Second, based on the envelope available and the vision for technical assistance, this can be short, medium, or longer-term. The duration is an essential factor in determining what support can be provided within the timeframe and resources available. For instance, vast change management processes would require at least medium-term engagement to allow for trust building between the advisor and the technical assistance recipient and to build capacity.

Third, the role of the technical advisors can differ depending on many factors. In practice, advisors would play one or more of the following roles:

- doers (substituting government capacity),
- partners (complementing government efforts and supporting them in areas of highly specialised expertise), or
- facilitators (supporting complex change programmes to strengthen state capability).

In terms of design, there is an *in-principle* agreement in the development community that problem-driven support is more effective than solution-driven technical assistance ( Sparrow, 2008), ( Andrews *et al*., 2015). However, the practice is diverse. Current technical assistance programmes employ a solution-driven (start with the solution and find ways to implement it) or a problem-driven approach (start with the problem and address the problem). The type of implementation of technical assistance can be different: for simple problems, traditional programme design can produce results following results frameworks with linear implementation and command and control type of management. For complex problems, a flexible framework of implementation is needed to allow learning, iterations, and a portfolio approach for problem-solving, while managing risks actively, focusing on results and failing forward. Some of the other types of the technical assistance are presented in Table 1.

**Table 1.** Types of technical assistance. The first column lists criteria for differentiation of technical assistance and the second column lists the different types of technical assistance.

Criteria	Types of technical assistance
Length of the engagement	• Short-term (a few months to 1 year) • Medium-term (1–3 years) • Long-term (3–5; 5–10 years)
Source of funding	• Technical advisers funded directly by sovereign governments • Technical advice publicly funded by donor organisations: • Multilateral organisations • Bilateral organisations • Supranational bodies • Technical advice funded by private organisations: • Philanthropies • (International) Non-Governmental Organisations
Type of funding	• Technical assistance tied to lending programmes and performance indicators • Technical assistance provided on a fee-for service (reimbursable advisory services) • Technical assistance supported through non-reimbursable funds • Grants to advisors, directed or not through the government
Objective	• Policy strengthening, along the policy cycle • State capability strengthening • Crisis response
Type of design	• Solution driven • Problem driven
Role of advisors	• Doers • Partners • Facilitators
Type of implementation	• Linear, command and control programme management • Adaptive • A mix of approaches
Type of staffing	• Mix of national and international consultants • International consultants • National consultants
Type of methods used	• Analytical support • Management support • Training and courses • Coaching • Facilitation • Mentoring • Peer exchange • On-the-job learning • Embedding external experts in the government
Counterparts	• Government departments • Civil society organisations • Coalitions across society • Local or regional networks • Decision-makers only
Level of support	• National/ federal level / central public institutions • Provincial level • Local level • Community level

Wanderman *et al*. looked at the growing evidence base for how technical assistance has improved outcomes in multiple contexts, based on four dimensions: dosage, mode of delivery, collaborative, and proactive design ( Wandersman *et al*., 2012). They found mixed results, indicating that technical assistance dosage is more likely to predict improvements in recent programmes and less likely in programmes that have run for a more extended period (Feinberg *et al*., 2008). A predictable finding in terms of mode of delivery shows that face-to-face interactions are more effective than remote interactions. Collaboration also seems to be an essential variable in influencing outcomes, mixing delivery approaches such as training, facilitated meetings and interpersonal exchanges ( Wesley & Buysse, 1996), as well as including stakeholders in the design (Spoth *et al*., 2017), and taking a broader view to the ecosystem ( Salyers *et al*., 2007).

**Figure 1.**
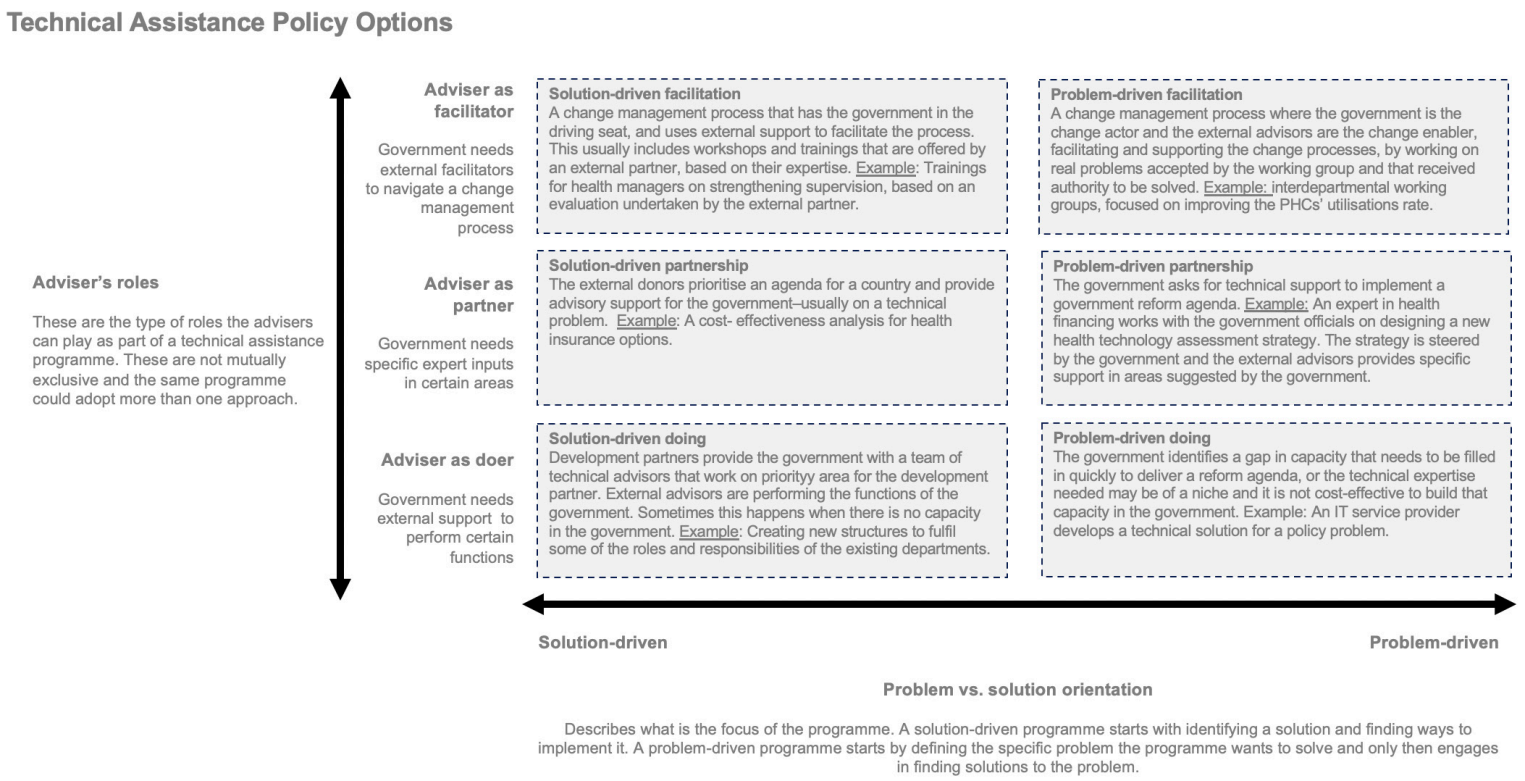
Technical assistance policy options. Each box represents a technical assistance model. Six boxes are presented on two axis – the adviser’s role and the problem orientation of the programme.

The framework presented in Figure 1 below refers to two of the most common features of technical assistance seen as a policy choice. This is not a normative framework guiding how externals should deliver technical assistance; it is an empirical framework for how capacity development is currently offered, focusing on the options governments now face ^1^. We refer to a matrix that differentiates between problem and solution orientation and roles for external advisors.

## 1. Problem or solution orientation

At the conceptual level, a solution-driven technical assistance programme would start by identifying an approach and advocate for its implementation in different contexts. By contrast, a problem-driven technical assistance programme would begin with the government or the donor defining the problem and only then moving to identifying and applying solutions.

Research shows that effective institutions are not developed by importing best international practices ( Johnson, 1982). The development community is rather unanimous in the rhetoric that a problem-driven approach is more appropriate to deliver technical assistance. The logical argument derives from the principles of national ownership (2005 Paris Declaration), according to which governments are best placed to identify their needs and problems to address, in accordance with their development priorities. Problems may be similar across geographies, but their operationalisation is different in each context. For instance, during COVID, countries started vaccination campaigns with vaccination being undertaken under similar if not identical protocols. However, the challenges to vaccination were very different, from supply to storage, lack of confidence in the vaccine, or in the administration of the vaccine etc.

In practice, many technical assistance and capacity development programmes have been solution-driven in the past decade, with development partners focused on solutions that can be applied across geographies.

Some of the possible explanations for the high frequency of solution-driven approaches are:

- **Path dependency** referring to governments receiving support for a long time based on adapting solutions from the global level hoping to produce similar results are less likely to change the type of TA without additional changes in their contexts or incentives structures.
- **Donors’ internal individual performance management criteria** do not include development results but include business development indicators meaning a more significant programme portfolio in-country government. In some cases, career progression within donor organisations depends more significantly on enlarging the country portfolio and than on the actual development that the programme has supported.
- **Lack of absorption capacity in the government institutions.** Even in cases where there is a will to improve the way institutions function or drive results in specific sectors, governments may face limitations in terms of how much capacity they have to absorb support that is not solution-driven. For instance, a problem-driven approach would require national counterparts to work on problem identification and analysis and to spend time on medium or longer-term reform agenda. In resource-poor environments, this is perceived as unrealistic.
- **The illusion of quick fixes.** Some wonder models for improving policymaking and delivering results quickly are marketed as a panacea to public sector change from time to time. Flashy solutions that can help politicians and high-level decision-makers show their commitment to reform are easily adapted across geographies.
- **Impatience.** To focus on problems, one needs to understand the context, understand the problem, and understand the stakeholders. This requires time, patience and political capital.
- **Lack of a culture of reflection.** The value of reflection is underestimated. Weak institutions are usually driven by a performance culture that derives from high-level decision-makers. In practice, this means that the day-to-day work is focused on the agenda of the superior. One of the disadvantages is that a high-level decision-maker agenda will almost always focus on urgent matters, changes quickly and ignores the values of reflection, lesson learning and long-term and less visible results.
- **Alleged government capacity to articulate problems.** Driving national reform through global definitions and frameworks can overwhelm the national institutions quickly, and sometimes either mute local voices or affect the capacity of governments to articulate their problems.

**Figure 2.**
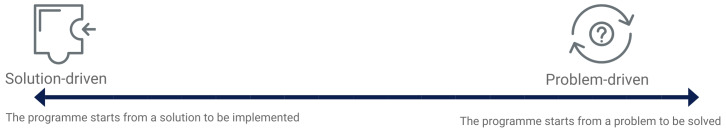
Problem orientation. Each icon represents a type of problem orientation, one is solution-driven and the other is problem-driven. The arrow shows that these may be placed on a continuum as part of the same programme and are, in practice, not mutually exclusive.

Figure 2 below shows that, in practice, the government may choose from a continuum of the problem and solution-driven approaches as part of the same technical assistance programme, depending on their needs.

## 2. Roles for the technical advisers

**Figure 3.**
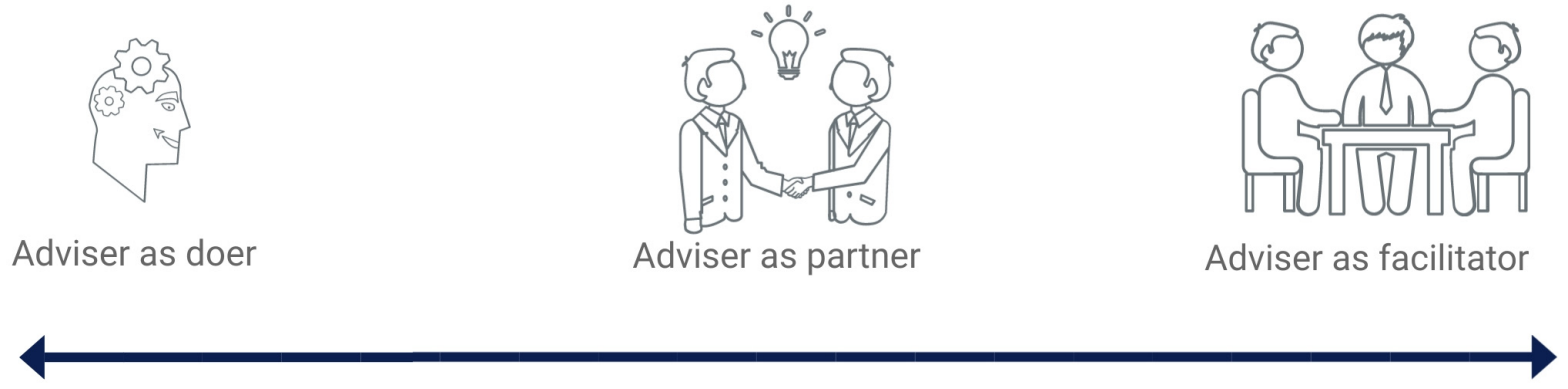
Adviser’s roles. Each icon represents a type of role for the technical advisers: doer, partner, facilitator. The arrow shows that these may be placed on a continuum as part of the same programme and are, in practice, not mutually exclusive.

Depending on the programme objectives, the role of technical advisers can be those of DOERS, PARTNERS, or FACILITATORS. The same team or the same programme may require a combination of different types of inputs, from short training sessions delivered by specialist consultants to change processes facilitated by externals that may require intensive engagement from the government and the externals, at multiple levels and over many years. In practice, the objectives may not be easy to isolate so we present the options on a continuum in Figure 3 below.

### 2.1. Adviser as doer

A standard model for the technical advisers is to be DOERS. For a variety of reasons, they perform government functions. This model is usually linked to capacity substitution or in-sourcing. At least two types of scenarios are seen in practice.

First, the government needs to perform specific functions but cannot perform them. This may refer to not having staffing or technical competencies. Sometimes, an urgent request and acquiring the inputs (mostly technical) needs to be expedited. At other times, the required capacity may refer to a niche that the government would not require beyond the current assignment, so they decide it is not good value for money to build the capacity in-house. For example, government agencies often rely on IT firms to develop internal systems and platforms –primarily one-off events – that are then delivered to and used by the host department.

Capacity substitution is usually deliberately applied in cases where:

•    the primary purpose of the support is not capacity development, but the delivery of specific predefined results;

•    there is an understanding of how the deliverables will fit into the broader system and an open channel to work with other government departments impacted by the work of the technical adviser; and/or

•    there is a lack of in-house technical expertise, needed urgently, or that may not make sense to develop in-house in the long run.

This model is frequently used in practice when the government or the donor is impatient to get results while cutting through red tape. As such, while there may be some value in this model for specific resourcing gap-filling needs, by design, this model has clear limitations in building state capability. Furthermore, the model can have severe consequences for state capability when the objectives are not clear. The technical advisors end up performing the core functions of the government, such as regulation, provision, funding, or service delivery. Table 2 below explores in detail the characteristics of this type of technical assistance, including its limitations.

**Table 2.** Three approaches to technical assistance.

	Capacity substitution	Capacity supplementation	Capacity development
**Role for the** **advisor**	DOER, performing government functions	PARTNER, providing specific support to the government (in most cases, technical support in specialised areas)	FACILITATOR, working with government to enable change and facilitate complex processes
**Policy need**	The government needs specific inputs but does not have the capacity to provide them – sometimes the need is urgent and no capacity is available, at other times the capacity needed is a niche that the government would not invest in beyond the current assignment so they in-source. • Clear technical problem • Known solution/output • Capacity constraints that cannot be filled with existing resources	The government needs specific expert inputs in certain areas. • Moderately sophisticated problems • Specific technical inputs required • Government requires additional support but leads the process	The government needs support in implementing a complex change process, and it requires specialised support to facilitate the process. • Complex change management problems • It is not clear what type of support is required • Support in identifying problems to solve, and testing different solutions, is required • The government will be involved and in the driving seat
**Programme** **characteristics**	• Clearly defined outputs to be delivered and reported against • Led by external actors with control from the government counterparts • Limited or no learning, the programme is mainly focusing on outputs • The advisors are expected to have technical competencies, and to be local or international experts.	• Predefined outputs but also outcome-based technical support • Led by the government counterparts or external actors • Learning depends on the context, the implementing partner, and it is not necessarily embedded in the programme design. • The advisors are expected to have both technical and professional skills, sometimes including facilitation skills in a specific area. In most cases, the expectation for the experts is to have international expertise.	• Outcomes-based programmes, with flexible and adaptive programme framework - regularly updated to navigate existing spaces for reform. • Led by the government and facilitated by external actors. • The role of the advisor is to manage a process, rather than the content of the programme. • Learning is central to programme design – both real-time learning for course correction as well as learning about what works, what doesn’t and why in that particular context. • The advisors can be both local and international, and the type of competencies required refer primarily to their facilitation skills and capacity to work with senior leaders and bureaucrats – although technical skills and experience in the area of work will be appreciated.
**Enabling** **conditions**	• Clearly identified gap in existing capacity • High levels of acceptance that there is a capacity gap to be filled • Some open channels to other government departments impacted by the work of the technical advisor • Authorisation to work on filling the capacity gap	• Medium levels of authority to engage on the subject • Medium levels of acceptance, ability, and willingness to partner with the advisor on the subject • An initial identification of areas of support needed	• High levels of acceptance that there is a problem to be solved/ something needs to be done • Authority, ideally from the highest political levels, to have the government teams engaged in the change process • At least medium level of ability of the government team to work on the change process
**Key risks**	• Lack of acceptance at various levels in the government (sometimes, a principal may request support but others in government may feel threatened or just not accept that the support is needed) • Lack of authority in government (the person who opts for this activity may lose authority or may not have authority over the relevant departments) • When this is based on supply, rather than demand, it may end up duplicating efforts and damaging the current reform efforts	• Lack of engagement from the government: sometimes the government may ask the advisor to lead the process, which eventually damages the level of ownership of the results • No authority in government • When this is based on supply rather than demand, it may provide highly technical advice, but not grounded in the local political economy • No follow-through after the end of the programme/ the funding period • No capacity developed, given a limited involvement from the government counterparts • Unintended consequences of diverting resources from other reform areas	• Having the support of a recognised authority in government is critical to undertake this type of activity. Relying too much on only one • person may damage the success of the programme • Losing the interest of the principal, for instance by focusing on too many long- term results and not enough quick wins to create acceptance • Focusing too much on developing technical skills and not understanding the political economy • Serious gaps in terms of hardware, infrastructure, supply chains, staff available • Unintended consequences of diverting resources from other reform areas

The methods of delivering this type of technical assistance vary. Sometimes programmes may involve niche technical support, such as building IT infrastructure; fellowships/consultancies of international or nationals working in host countries to deliver independently on behalf of the government; or reports, strategies etc. prepared by the external experts with no involvement from the government staff.

### 2.2. Adviser as partner

This model is usually linked to capacity supplementation. However, the government needs are also primarily technical and relate to the previously identified gap areas. The premise is that the government is already performing certain functions but requires specific inputs in certain challenging areas or needs to bring in newer and better ways of working from the outside. This model of technical assistance is deliberately applied in cases where:

•    the primary purpose of the support is to deliver outputs, and transfer knowledge to the host government.

•    the need for support is limited and clearly identified, and the technical assistance providers can bring in that specific expertise; and/or

•    the government is looking for support in specific challenging areas, and they are leading the programme implementation.

Many small to medium-sized development projects fall under this category. Governments make the best use of this type of support when it is demand-driven and when the inputs required from the externals specifically contribute to solving a problem they are tackling. For example, in certain Indian states, the departments of environment and climate change bring in local academic institutions and technical firms to execute highly specialised work, such as co-developing international funding proposals for climate adaptation projects. Similarly, multiple state-level departments of an industry often engage professional consulting firms to support their private investment promotion wings and bring more professional and corporate ways of working, which can work better with potential private investors.

The methods of delivering this type of technical assistance vary. Most frequently, this includes delivering outputs such as studies, reports, strategies, analysis by working with government counterparts. It can also include fellowships – when the fellows are working with governments on a specific project for a set timeframe. Often, external technical assistance aimed at capacity supplementation might need to demonstrate the effectiveness of the new ways of working and/or innovations being introduced to the government by ‘doing’ them themselves. However, this activity’s temporary or tactical nature distinguishes this type of technical assistance from the previous type (substitution/in-sourcing).

### 2.3. Adviser as facilitator

There seems to be a consensus that for technical assistance focused on behaviour and systems change, a strongly facilitator-based approach is necessary ( Le **et al*.,* 2016). Models of technical assistance that explicitly build long-term capacity in the government rely heavily on having the government counterparts in the driving seat and the advisor as merely a facilitator and change manager. While such a model can be used for reform processes of varying complexity, it is most valuable when embarking on sophisticated and complex change processes. The role of the facilitator is usually to help the government navigate the various stages of the change management process and challenge the way of doing things.

This model of technical assistance is usually applied in cases where:

•    the primary purpose of the support is to develop long-lasting capacity at individual, organisational, and institutional levels in the host government.

•    the government and the development partner are ready to invest time in building capacity sustainably – it will take longer to see the result. Still, there is a higher chance that these will be sustainable.

•    the need for support relates to change-facilitation, or behaviour and systems change, and the specific outcomes or change pathways are not clearly defined or identified.

•    the nature of the problem is highly dynamic, fluid, and ever-changing and requires significant changes in the hardware and software elements of organisations. ( Sheikh *et al*., 2011).

•    the process for reaching the result is not always clearly defined; he government cannot exercise full control over the reform process as there are many systemic challenges that influence the reform process.

Multiple methods have been applied to develop this type of approach in the past few years, including: problem-driven iterative adaptation (facilitating a process through which the government team engages in solving problems), mentorship (using recognised external experts in particular fields to guide some development objectives), and coaching (using external coaches to help individuals achieve the objectives, by providing a motivating environment and by challenging current ways of working, thinking etc). Fellowships can also be found in this category if they work with nationals and focus on supporting their development as part of the systems. Since the typical view of technical assistance supporting the government is long term, structural reforms in core public delivery processes and sub-systems are attempted in this type of assistance (e.g., public financial management systems, data/management information systems, performance management systems, and human resources policies).

## The implications of these policy options for the programming led by development partners

In Table 2, we captured the main features of technical assistance based on the roles played by the technical advisers. This framework is helpful for a few reasons:

- it provides an understanding of the advantages and limitations of playing each type of role.
- it helps decision-makers make informed decisions about what each type of role can realistically achieve by design.
- it supports a conversation within donor organisations, implementers and other technical advisers on their impact model based on methods of delivering support.

Further, two conversations are vital based on this framework. The first one refers to the expectations from technical assistance regarding capacity development. The second one refers to transitions between different roles for technical advisers.

### Expectations from technical assistance regarding capacity development

The most common fallacy of technical assistance programmes is to expect every type of technical assistance to lead to capacity development. By ‘ ***capacity development’*** we refer to enabling national actors to deliver functions they are designed to deliver. Building capacity usually takes time, patience, resources, consistency, and complementarity. It goes beyond the life of one programme and can require donors to come together in a joint effort to support the country’s development objectives.

First, governments need to balance the short-term political agenda with the long-term institutional development agenda. Public life is characterised by short-termism, especially in low-trust environments ( OECD, 2013). In programming terms, this balancing act translates into a tension between getting results done quickly on targets accessible to the citizens and building long-term capacity in institutions meant to deliver those services. When resources are limited, including time and political capital, decision-makers generally choose the quick wins and leave the institutional development agenda for a later stage.

Second, other development partners have their own pressures to show results quickly. Donors need to show results in using taxpayer money; the implementer needs to show results to the donor for the annual appraisals. This may result in less patience to focus on capacity development and processes and more DOING than FACILITATING.

Third, governments need to balance their needs in terms of capacity. Sometimes they need specialised support to solve a technical problem (which we termed capacity substitution). At other times, they may need high-level challenging functions to support them to achieve their objectives (which we termed capacity development). With clarity about the merits and limits of each approach, a large multi-year technical assistance programme for system strengthening may successfully draw on different types of support at various times.

Fourth, the accepted rhetoric and the real-life practices have diverged in the past few years. Currently, it is not acceptable for a development partner to publicly recognise that they are doing capacity substitution, even in the most resources-deprived environments. The rhetoric only praises the adaptable programmes that engaged on a system-level with traction across levels of governments and other relevant stakeholders in the community. What happens to those programmes running for a long-time, which have gradually substituted government functions and are struggling to change their delivery model – sometimes because their withdrawal would mean the collapse of an entire public service delivery system?

Not being able to talk openly about how those programmes have justified their interventions in the first place, what they achieved despite their capacity substitution model, and their challenges perpetuates a somehow incomplete picture of how development works. Conversations about these programmes should start with clear definitions of the type of capacity support – in this case, capacity substitution, not capacity development – and the solutions to breaking this cycle of a DOER approach. 

Fifth, the balance of power between governments and donors also influences capacity development programmes. The more invested the donors are, the more power they can have in influencing the reform agenda. In cases where they provide substantial financial support to national reform programmes, it may be difficult for some government counterparts to negotiate their need for technical assistance. The most daunting consequence may be for government counterparts to feel disempowered in making technical assistance choices instead of seeing this as a building block to accessing the required financial support.

Government, funders, and technical assistance providers must ensure that due attention is invested in building this shared (and honest) view of the problems that the support is seeking to tackle. Ring-fencing these issues during the design stage, and subsequently developing and agreeing on the appropriate rules of engagement between the parties, is key to ensuring that the support remains focused on the core issues and can build sustainable capacity in the recipient government over time. This process requires a meaningful and equal dialogue between governments and funders in the design of technical assistance programmes.

Sixth, building the right teams to deliver capacity development requires some changes in how development partners deploy staff. Historically, technical skills have been prioritised above other skills, such as interpersonal skills, understanding of political context, and relatable expertise. As the role of technical assistance providers gradually shifts from pure implementation to more facilitation, it will be essential to engage individuals who play that convening role and technical leadership. It is important to engage advisors who can build trust with counterparts and communicate and network effectively. At the same time, power may need to shift from international to national experts. This is easier said than done. A few challenges may need to be overcome. For instance, the current power structures favour international technical experts, including donor organisations and implementing partners. Additionally, there is an accountability trap of focusing on high-quality results and less on building processes to support capacity development in the government and the larger ecosystem – including local consultants and external local organisations, who are likely to be present there after the end of the support programme.

### Transitions between different roles for technical advisers

Some technical assistance programmes use a theory of change that proposes a model of technical assistance starting with DOING, gradually transitioning to PARTNERING, and to a FACILITATION role. The main assumption for these programmes is that handholding along this process will achieve results gradually. More research is needed to test the validity of this assumption. We see two main challenges.

Managing expectations upfront about the transition is critical. Starting the programme with a plan to make yourself irrelevant (as a donor, as an implementing partner) is central. The problem is that usually, the transition plans are prepared towards the end of the programme, which creates difficulties in successfully undertaking the transition. One reason is that the necessary people, processes, and relationships differ when performing different roles (doer, partner, facilitators). This leads to situations in which transitions seem artificial and cannot lead to sustainable changes – a long-time DOER will find it difficult to become a FACILITATOR after engaging with the government for a long time. Sometimes, this shift may also be perceived as withholding resources from the government, which will increase reluctance to change.

More importantly, the transition urgency needs to come from the government. Donors can, of course, initiate or advocate for it, but successful transitions need country ownership. Research on the successful cases of Botswana and Mauritius shows that stable environments, with political actors committed to institutional reform have ensured the premises the country’s economic development. ( Kiiza, 2006).

## The opportunities for change

COVID-19 has exacerbated some of the challenges in the design and implementation of technical assistance, with a particular focus on local ownership and delivery and the urgency to build resilient and adaptive state capability that supports public service delivery.

### Local ownership and delivery

Even before the pandemic, there was an increasing demand from national governments to provide national or regional expertise, a growing recognition from development partners that context does matter, and that national and regional knowledge is critical to success. We can see this shift in the increasing focus of UK development projects on national and regional experts and in Australia’s strategy on localising aid ( Cornish, 2019). As highlighted before, the public rhetoric supports a rebalancing of how valuable local or international support is. The remaining agenda is to provide tangible proof of the commitment to valuing local expertise, through remuneration according to expertise and contribution comparable to international standards.

In the past two years, with limited possibilities for international travel, the options to deliver technical assistance were also finite. This has forced both providers and funders of technical assistance to adapt their delivery model and ensure local ownership and delivery. As a result, the years 2020-2021 were characterised by a need for rapid support in key areas of technical expertise, from understanding how to manage fiscal space to allocate funds and manage the crisis to building massive social assistance programmes to attenuate the immediate shocks on households and the economy. From our experience, we have seen results of timely and useful technical assistance in countries where capable, empowered local teams were already in place, and used to a decentralised decision-making model. These teams delivered the most useful work to the governments, while also balancing the urgency of the crisis with transformational work ( Rajadhyaksha *et al*., 2020).

Change in the way technical assistance is delivered, and the emphasis on local or international experts has been slowed down by reasons that have to do primarily with managing risks, including strategic, operational, reputational, quality, and most importantly fiduciary risks ( French & Nastase, 2020). This connected financial assistance to technical assistance, despite the lack of solid evidence for this connection ( Teskey, 2020). At the core of risk management for large ambitious and adaptable programmes is a well-thought credible governance framework. This provides reassurance to donors that taxpayers’ money is spent for achieving valuable outcomes, with all due diligence required. There is a growing learning base documenting various practices in setting up adaptive and locally-led programmes that practitioners can access ( Cooke, 2017; Laws *et al*., 2021)

Stricter codes of conduct to support problem definition as a locally led exercise are also needed. The lack of consequences when ignoring aid effectiveness principles incentivises a vicious circle in which technical assistance does not respond to real country needs and gradually erodes national capacity and credibility of development efforts overall.

Local actors need to be involved beyond the design stages or beyond consultations. More importantly become a part of the national accountability mechanisms and get involved in all stages of the programme implementation.

### Urgency to build resilient and adaptive state capability

Most countries today experience significant fiscal pressure. As a result, low-income countries will need support, financial assistance ( Henstridge, 2020). This will create an opportunity for policy dialogue between national governments and development partners. Ideally, governments will consider technical assistance as a policy option. The guiding principle is to choose technical assistance aligned with the country’s development priorities while fully understanding the advantages and limitations of doing, partnering, or facilitating change ( Nastase *et al*., 2020), gathering evidence about what works, and implementing programmes using adaptive practices that are both focusing on solving problems and learning about what works.

During COVID-19, decision-makers in governments have been forced to make decisions to address the effects of the pandemic with unclear scientific evidence of what works and need to revisit those decisions quickly to account for relevant new information ( Akroyd *et al*., 2020). Adaptive capacities require, among others, being open and transparent about learning, using collective decision-making process and building trust with communities and individuals ( Ramalingam *et al*., 2020). However, responding to countries’ needs to develop adaptive capabilities also needs to be followed by donors’ rules and procedures on how support is procured and how success is measured. While the demand for more innovative, adaptative programmes is increasing, the systems of procurement and evaluation have not entirely followed through, which inhibits innovation and the focus on outcomes.

### Honest conversations about the future

Open conversations need to happen within the donor organisations where the commitment to achieving SDG and supporting countries’ development priorities need to influence internal performance management structures. As long as individual career progressions within donor organisations depend more on business development success than on enabling sustainable reform in recipient countries, technical assistance will only suffer.

Additionally, these conversations about the current roles technical advisors play, their limitations, but also advantages are important to managing expectations about how to support governments better. This would also imply a departure from calling everything ‘capacity development’ and from agreeing, in principle to ambitious agenda to reimagining technical assistance without following through with palpable commitments to respecting country ownership, valuing local expertise, and spending time to understand context before proposing ‘bullet proof’ solutions to public sector management issues.

## Data and availability

No data are associated with this article.

## References

[ref-10] Akroyd S Harrington P Nastase A : Rapid Literature Review – Governance and State Capability.2020. Reference Source

[ref-1] Andrews M Pritchett L Woolcock M : Escaping Capability Traps through Problem Driven Iterative Adaptation (PDIA) - CID Working Paper No. 240.Boston: Center for International Development, Harvard University.2012. Reference Source

[ref-11] Andrews M Pritchett L Woolcock M : Doing Problem Driven Work.CID. Working Paper, 307. Center for International Development at Harvard University,2015. 10.2139/ssrn.2700308

[ref-12] Andrews M Pritchett L Woolcock M : Building state capability. Evidence. Analysis. Action.Oxford University Press,2017. Reference Source

[ref-2] Cashin C : The Coaching Approach: Changing the way development assistance is done.Online:2020; Accessed on September 2020. Reference Source

[ref-13] Cooke K : How to set up and manage an adaptive programme. Lessons from the Action on Climate Today (ACT) Programme.Oxford Policy Management,2017. Reference Source

[ref-3] Child Health Task Force: Reimagining Technical Assistance. Nigeria Status Update. 2019. Reference Source

[ref-14] Cornish L : Making Australia’s humanitarian assistance fit for the future.2019. Reference Source

[ref-4] Faustino J Booth J : Development entrepreneurship – how donors and leaders can foster institutional change. 2014. Reference Source

[ref-15] French B Nastase A : Reimagining technical assistance: Will COVID-19 change delivery.2020. Reference Source

[ref-16] Henstridge M : The impact of COVID-19 pandemic on low - and middle- income countries.Oxford Policy Management.2020. Reference Source

[ref-18] Johnson C : MITI and the Japanese Miracle: The growth of industrial policy, 1925-1975.Stanford, Stanford University Press,1982. Reference Source

[ref-17] Kiiza J : Institutions and Economic Performance in Africa. A Comparative Analysis of Mauritius, Botswana and Uganda.The United Nations University. UNU-World Institute for Development Economics Research. Research paper No. 2006/73. Reference Source

[ref-19] Laws E Pett J Proud E : LearnAdapt: A synthesis of our work on adaptive programming with DFID/FCDO (2017-2020).2021. Reference Source

[ref-5] Laws E Marquette H : Thinking and working politically. Reviewing the evidence on the integration of politics into development practice over the past decade. 2018. Reference Source

[ref-6] Le LT Anthony BJ Bronheim SM : A Technical Assistance Model for Guiding Service and Systems Change. *J Behav Health Serv Res.* 2016;43(3):380–95. 10.1007/s11414-014-9439-2 25239308

[ref-20] Nastase A Rajan A French B : Technical assistance: a practical account of the challenges in design and implementation [version 1; peer review: 1 approved, 1 approved with reservations]. *Gates Open Res.* 2020;4:177. 10.12688/gatesopenres.13205.1 35299599 PMC8920999

[ref-21] OECD: Government at a Glance.2013. 10.1787/22214399

[ref-22] Rajadhyaksha M Nastase A Khan M : COVID-19 and the politics and policymaking.2020. Reference Source

[ref-23] Ramalingam B Wild L Ferrari M : Adaptive leadership in the coronavirus response Bridging science, policy, and practice.Overseas Development Institute,2020. Reference Source

[ref-24] Salyers MP McKasson M Bond GR : The role of technical assistance centers in implementing evidence-based practices: Lessons learned. *Am J Psychiatr Rehabil.* 2007;10(2):85–101. 10.1080/15487760701345968

[ref-25] Sheikh K Gilson L Agyepong IA : Building the Field of Health Policy and Systems Research: Framing the Questions. *PLoS Med.* 2011;8(8):e1001073. 10.1371/journal.pmed.1001073 21857809 PMC3156683

[ref-26] Sparrow MK : The character of harms: Operational challenges in control.Cambridge: Cambridge University Press,2008. 10.1017/CBO9780511753862

[ref-27] Teskey G : Covid + w@h + Zoom = a big change for TA and Managing Contractors?2020. Reference Source

[ref-8] TWP CoP: So what does ‘thinking and working politically’ look like? 2013. Reference Source

[ref-9] USAID: ADS Chapter 201 - Program Cycle Operational Policy. 2016. Reference Source

[ref-28] Wandersman A Chien VH Katz J : Toward an evidence-based system for innovation support for implementing innovations with quality: tools, training, technical assistance, and quality assurance/quality improvement. *Am J Community Psychol.* 2012;50(3–4):445–59. 10.1007/s10464-012-9509-7 22538406

[ref-29] Wesley PW Buysse V : Supporting early childhood inclusion: Lessons learned through a statewide technical assistance project. *Topics Early Child Spec Educ.* 1996;14(4):476–500. Reference Source

