# Supplementary material for: Towards reimagined technical assistance: the current policy options and opportunities for change
Source: Gates Open Res. 2021 Oct 18;4:180. Originally published 2020 Dec 16. [Version 2] doi: 10.12688/gatesopenres.13204.2 (PMC10957524; doi:10.12688/gatesopenres.13204.2)
Supplement: Peer review history

# Reviewer report: Reviewer response for version 2

**DOI:** 10.21956/gatesopenres.14631.r36042

**Published:** 2024-07-22

**Authors:** Wilson Wong

**Affiliations:** 1 The Chinese University of Hong Kong, Hong Kong, Hong Kong

This is an outstanding article that integrates various approaches and theories of technical assistance to formulate a comprehensive model. The model successfully bridges academic theories and practical experience, providing valuable guidance for policymakers. It identifies problems and challenges while offering suggestions on how to address and overcome them, making technical assistance a viable policy option. The article compellingly argues, with both theoretical and empirical justifications, that the most desirable approach is problem-orientation rather than solution orientation and emphasizes the role of the advisor as a facilitator to promote capacity building.

 While the article exhibits strengths, it could be further enhanced by incorporating insights and perspectives from broader interdisciplinary approaches. As a scholar specializing in comparative public policy and governance, I recommend the authors to consult relevant literature in the following areas. Key references for each area are provided at the end of this review.

 Firstly, collaborative governance is defined as "the processes and structures of public policy decision making and management that engage people constructively across boundaries." (Emerson et al., 2012) [Ref 2]. Since technical assistance involves building enduring relationships between international experts and local governments, collaborative governance offers valuable knowledge on the subject. In addition to Emerson et al. (2012), the authors can consult Ansell & Gash (2008)[Ref 1] for a gateway to significant research and literature on collaborative governance, a core area of public administration research in recent decades.

 Secondly, the article should exercise caution and distinction when using the concept of state capacity. Differentiating between state capacity and government capacity is crucial. If the technical assistance aims to enhance the capacity of local public agencies and the executive branch, government capacity is the appropriate term. If the focus is state capacity, the article should also discuss how institutions outside the executive branch can be strengthened for a holistic view. At the same time, the capacity of civil society should also be considered in order to understand the full policy capacity of a country. In other words, empowering civil society can be as important as strengthening governments. Moon & Ingraham (1998) [Ref 10], Moon et al. (2021) [Ref 4], Wong & Wu (2022) [Ref 14]provide valuable research on this topic.

 Thirdly, the discussion on the impact of COVID on technical assistance can be generalized to emphasize the critical role of crises as policy windows for permanent institutional changes. Policy entrepreneurs play a vital role in overcoming resistance and self-interest, converting window opportunities into results. The "multiple streams framework" (MSF) encompasses the roles, interactions, and impacts of crises and policy entrepreneurs in effecting policy change (see Kingdon & Stano, 1984 [Ref 5]; Mintrom & Norman, 2009) [Ref 9], which is relevant to this discussion.

 Lastly, when discussing technical transfer which often involves knowledge and technology, it is essential to adopt a broader perspective and consider contextual factors for capacity building and institutional changes. It has been well documented in the literature of technology studies that successful technical assistance goes beyond knowledge and technology transfer; it includes organizational and institutional transformation. The article can benefit from referring to literature on information and communication technologies (ICT), including the latest developments in AI, as well as administrative reforms (see Fountain, 2004 [Ref 3]; Kraemer & King, 2006 [Ref 6]; Liu & Yuan, 2015 [Ref 7]; Luna-Reyes & Gil-Garcia, 2014 [Ref 8]; Tornatzky & Fleischer, 1990 [Ref 11]; Van Noordt & Misuraca, 2022 [Ref 12]; Wong & Welch, 2004)[REf 13]for additional insights and details.

Does the article adequately reference differing views and opinions?

Partly

Are all factual statements correct, and are statements and arguments made adequately supported by citations?

Yes

Is the Open Letter written in accessible language?

Yes

Where applicable, are recommendations and next steps explained clearly for others to follow?

Yes

Is the rationale for the Open Letter provided in sufficient detail?

Yes

Reviewer Expertise:

Public Policy, Public Administration, Public Governance, Comparative Studies, ICT and AI

I confirm that I have read this submission and believe that I have an appropriate level of expertise to confirm that it is of an acceptable scientific standard.

## References

[1] : Collaborative Governance in Theory and Practice. *Journal of Public Administration Research and Theory* .2008;18(4) : 10.1093/jopart/mum032 543-571 10.1093/jopart/mum032

[2] : An Integrative Framework for Collaborative Governance. *Journal of Public Administration Research and Theory* .2012;22(1) : 10.1093/jopart/mur011 1-29 10.1093/jopart/mur011

[3] : Building the Virtual State. *https://www.brookings.edu/books/building-the-virtual-state/* .2001;

[4] : A comparative study of COVID-19 responses in South Korea and Japan: political nexus triad and policy responses. *International Review of Administrative Sciences* .2021;87(3) : 10.1177/0020852321997552 651-671 10.1177/0020852321997552 PMC868556440479109

[5] : Agendas, alternatives, and public policies (Vol. 45, pp. 165-169). *https://search.worldcat.org/title/Agendas-alternatives-and-public-policies/oclc/10277820* .1984;

[6] : Information Technology and Administrative Reform. *International Journal of Electronic Government Research* .2006;2(1) : 10.4018/jegr.2006010101 1-20 10.4018/jegr.2006010101

[7] : The Evolution of Information and Communication Technology in Public Administration. *Public Administration and Development* .2015;35(2) : 10.1002/pad.1717 140-151 10.1002/pad.1717

[8] : Digital government transformation and internet portals: The co-evolution of technology, organizations, and institutions. *Government Information Quarterly* .2014;31(4) : 10.1016/j.giq.2014.08.001 545-555 10.1016/j.giq.2014.08.001

[9] : Policy Entrepreneurship and Policy Change. *Policy Studies Journal* .2009;37(4) : 10.1111/j.1541-0072.2009.00329.x 649-667 10.1111/j.1541-0072.2009.00329.x

[10] : Shaping Administrative Reform and Governance: An Examination of the Political Nexus Triads in Three Asian Countries. *Governance* .1998;11(1) : 10.1111/0952-1895.581998058 77-100 10.1111/0952-1895.581998058

[11] : The processes of technological innovation. Lexington Books. *https://www.researchgate.net/publication/291824703_Technological_Innovation_as_a_Process* .1990;

[12] : Exploratory Insights on Artificial Intelligence for Government in Europe. *Social Science Computer Review* .2022;40(2) : 10.1177/0894439320980449 426-444 10.1177/0894439320980449

[13] : Does E‐Government Promote Accountability? A Comparative Analysis of Website Openness and Government Accountability. *Governance* .2004;17(2) : 10.1111/j.1468-0491.2004.00246.x 275-297 10.1111/j.1468-0491.2004.00246.x

[14] : State or Civil Society – What Matters in Fighting COVID-19? A Comparative Analysis of Hong Kong and Singapore. *Journal of Comparative Policy Analysis: Research and Practice* .2022;24(6) : 10.1080/13876988.2021.1978819 609-626 10.1080/13876988.2021.1978819

# Reviewer report: Reviewer response for version 2

**DOI:** 10.21956/gatesopenres.14631.r36041

**Published:** 2024-03-21

**Authors:** Gabriel Lele

**Affiliations:** 1 Universitas Gadjah Mada Indonesia, Region of Yogyakarta, Indonesia

This article is well written, displaying the models of technical assistance with its advantages and disadvantages. The writers compare and analyze the models and present recommendation for technical assistance improvement. If there is one area to be improved, it is concerning the reference to differing views. The authors may need to elaborate a little bit deeper on the advantages and disadvantages of each model. The authors may also need to present a more applicable recommendation on which model to follow and the preconditions for such model. Moreover, in analyzing the models and proposing recommendations, the authors may need to use legitimacy and accountability as two additional working principles other than ownership.

Does the article adequately reference differing views and opinions?

Partly

Are all factual statements correct, and are statements and arguments made adequately supported by citations?

Yes

Is the Open Letter written in accessible language?

Yes

Where applicable, are recommendations and next steps explained clearly for others to follow?

Yes

Is the rationale for the Open Letter provided in sufficient detail?

Yes

Reviewer Expertise:

Global Public Policy, Decentralization, and Good Governance

I confirm that I have read this submission and believe that I have an appropriate level of expertise to confirm that it is of an acceptable scientific standard.

# Reviewer report: Reviewer response for version 1

**DOI:** 10.21956/gatesopenres.14414.r30615

**Published:** 2021-05-20

**Authors:** Jason Katz

**Affiliations:** 1 Domestic Capacity Building Service Area, American Institutes for Research, Washington, DC, USA

To advance development outcomes, this article frames technical assistance (TA) as a policy option and provides an actionable framework for classifying types of technical assistance (TA) according to different roles for TA providers in related to government.

- The introduction would benefit from examples of development outcomes that can be enhanced via TA in a theory of change. Also, a chart would be useful that highlights the relevant features of the referenced TTA approaches.
- The second section also requires more justification and background. For example, I wasn’t clear about the statement “the same type of support is available no matter who finances it.”  
- The framework presented in Figure 1 is very helpful. An apparent alternative is to start with needs/problems and select solutions in light of those needs and resources – for example, this is the approach of Getting to Outcomes:
  - Wandersman *et al.* (2012 ^1^).
  - Katz and Wandersman (2016 ^2^).
- The authors may want to weave this into this discussion.
- The article outline needs work – the headings and the organization was sometimes confusing. For example, why isn’t there a section on the solution orientation? A good copy edit would also be helpful.

Overall, this article is very good thinking and will be an important contribution if some of the technical issues mentioned above are addressed.

Does the article adequately reference differing views and opinions?

Yes

Are all factual statements correct, and are statements and arguments made adequately supported by citations?

Partly

Is the Open Letter written in accessible language?

Partly

Where applicable, are recommendations and next steps explained clearly for others to follow?

Yes

Is the rationale for the Open Letter provided in sufficient detail?

Partly

Reviewer Expertise:

Implementation science

I confirm that I have read this submission and believe that I have an appropriate level of expertise to confirm that it is of an acceptable scientific standard, however I have significant reservations, as outlined above.

## References

[1] : Toward an evidence-based system for innovation support for implementing innovations with quality: tools, training, technical assistance, and quality assurance/quality improvement. *Am J Community Psychol* .2012;50(3-4) : 10.1007/s10464-012-9509-7 445-59 10.1007/s10464-012-9509-7 22538406

[2] : Technical Assistance to Enhance Prevention Capacity: a Research Synthesis of the Evidence Base. *Prev Sci* .2016;17(4) : 10.1007/s11121-016-0636-5 417-28 10.1007/s11121-016-0636-5 26858179 PMC4839040

# Response

**Published:** 2021-09-07

**Authors:** Alexandra Nastase

**Affiliations:** Oxford Policy Management, UK

Thank you for taking the time to review our article. We have now revised the document in line with the suggestions received.

 Please find below our responses:

- We included the references to how TA enhances development outcomes. The suggested articles included useful research. Thanks!
- We also included a chart for types of TA in the complementary open letter that can be accessed here: https://gatesopenresearch.org/articles/4-180/v1
- Thanks for noting the lack of clarity, we rephrased this accordingly
- We updated the structure and the headlines and copy edited the article again.
- Thank you for the suggested literature. We read and included the references in our bibliography.

We hope this responds satisfactorily.

# Reviewer report: Reviewer response for version 1

**DOI:** 10.21956/gatesopenres.14414.r30153

**Published:** 2021-02-09

**Authors:** Ligia Paina

**Affiliations:** 1 Department of International Health, Johns Hopkins Bloomberg School of Public Health, Baltimore, MD, USA

Thank you for the opportunity to review. This is a well-written article, framing technical assistance as a policy option - a unique and important topic.

 I propose three suggestions for the authors' consideration:

1. To address the power issues in technical assistance more deeply, also to recognize that there are imbalances between providers of technical assistance and government counterparts, and not just between governments and donors.
2. To distinguish between short-term and long-term engagements in technical assistance and the pros and cons of each.
3. To reflect on the emerging literature on decolonization and to reflect on the future role of technical assistance in this context. 

Does the article adequately reference differing views and opinions?

Yes

Are all factual statements correct, and are statements and arguments made adequately supported by citations?

Yes

Is the Open Letter written in accessible language?

Yes

Where applicable, are recommendations and next steps explained clearly for others to follow?

Yes

Is the rationale for the Open Letter provided in sufficient detail?

Yes

Reviewer Expertise:

health systems research, health policy analysis

I confirm that I have read this submission and believe that I have an appropriate level of expertise to confirm that it is of an acceptable scientific standard.

# Response

**Published:** 2021-09-07

**Authors:** Alexandra Nastase

**Affiliations:** Oxford Policy Management, UK

Thank you for taking the time to review our article. We have now revised the document in line with the suggestions received.

 Please find below our responses:

- On the issues of power, we tried to give it more space in the article, building on both the existing literature and our experience.
- We added a table that discusses the types of TA based on the length of TA. We also included notes about dependency on the length of the engagement and the type of capacity that can be provided.
- We added references to decolonizing aid and to further localizing it. We also added a section on opportunities for change, especially in the context of COVID -19 pandemic.

We hope this responds satisfactorily.
